# Impact of aging on the central and enteric nervous system in a Parkinson’s disease mouse model

**DOI:** 10.3389/fnagi.2025.1576325

**Published:** 2025-12-01

**Authors:** Solène Pradeloux, Morgane M. André, Maélie Fréchette, Mathilde Drolet, Catherine Fontaine-Lavallée, Mélissa Côté, Katherine Coulombe, Marie Rieux, Frédéric Calon, Denis Soulet

**Affiliations:** 1 Centre de Recherche du CHU de Québec, Québec, QC, Canada; 2 Faculté de Pharmacie, Université Laval, Québec, QC, Canada

**Keywords:** Parkinson’s disease, inflammaging, enteric nervous system, central nervous system, MPTP mouse model, myenteric plexus, submucosal plexus

## Abstract

The etiopathogenesis of Parkinson's Disease (PD) remain poorly understood, particularly the roles of aging and the gut-brain axis. This study investigated the impact of aging on the development of PD hallmarks, including neurodegeneration and inflammation, in both the central (CNS) and enteric (ENS) nervous system of mice following exposure to 1-methyl-4-phenyl-1,2,3,6-tetrahydropyridine (MPTP). Young (2-4 months) and adult (7-12 months) mice were treated with either saline or MPTP (four intraperitoneal injections of 8 mg/kg at 2-hour intervals). Postmortem inflammatory and neuronal endpoints were compared in both the nigrostriatal pathway and the myenteric plexus. While age did not alter the MPTP-induced reduction in TH-positive cells in the striatum and substantia nigra pars compacta (SNpc), we observed a greater sensitivity of enteric DAergic neurons to MPTP neurotoxicity with age. Notably, MPTP treatment elicited a more prominent inflammatory response in the SNpc and the myenteric plexus in older animals, as assessed with Iba1 and GFAP immunofluorescence on brain sections. We also observed enteric and central inflammation, an increase in oxidative stress in the SNpc measured with Nrf2, and a loss of enteric DAergic neurons with aging, comparable to what is observed in young mice treated with MPTP. The enhanced vulnerability of the ENS is consistent with the observation that intestinal symptoms precede motor symptoms in PD, suggesting that immunosenescence in the gastrointestinal tract contributes to the early development and progression of PD.

## 1 Introduction

Parkinson’s disease (PD) is the second most common neurodegenerative disease, affecting approximately 3% of the population over the age of 65 and increasing to 5% of people over the age of 85. This prevalence is likely to increase due to the aging population, with age being the primary risk factor for PD (Lee and Gilbert, 2016; Savica et al., 2016; Tysnes and Storstein, 2017; Blauwendraat et al., 2019). It is a progressive neurological disorder characterized by the selective degeneration of dopaminergic (DAergic) neurons and their projections in the nigrostriatal pathway, leading to the development of motor dysfunctions (tremors, bradykinesia, postural instability, muscle rigidity) that allow the diagnosis of PD (Klingelhoefer and Reichmann, 2015; Sveinbjornsdottir, 2016). Another neuropathological hallmark of PD is the presence of intracellular inclusions named Lewy bodies, mainly composed of alpha-synuclein (*α*-Syn) (Braak et al., 2003a; Berg et al., 2014). Inflammation is also an important factor contributing to neuronal degeneration in PD initiation and development. Notably, hyperactivated microglia and reactive astrocytes are present in the substantia nigra *pars compacta* (SNpc) and *caudate putamen* of PD patients (McGeer et al., 1988; Kohutnicka et al., 1998; Halliday and Stevens, 2011). In addition to pro-inflammatory cytokines found in the nigrostriatal pathway and cerebrospinal fluid, peripheral immune cells are also altered in patients (Mogi et al., 1994; Nagatsu et al., 2000). Microglia can participate in neuronal cell death by aberrant engulfing of neurons and production of inflammatory mediators, thus accelerating PD progression (Mogi et al., 1994; Ouchi et al., 2009; Nayak et al., 2014).

Physiological aging in humans is characterized by a progressive decline in motor skills and neuronal degeneration in the brain, mirroring key features of PD, without producing clinically relevant PD signs (Volkow et al., 1998; Calabrese et al., 2018). Age not only influences the onset and progression of PD but also presents distinct phenotypes of the disease (Hely et al., 1995). Nigrostriatal system dysfunction occurs during normal aging, with regional patterns similar to those seen in PD (Irwin et al., 1994; Chu and Kordower, 2007; Kanaan et al., 2008). Cellular mechanisms contributing to impaired neuronal function during aging are also present in PD, such as mitochondrial dysfunction, inflammation, oxidative stress, and impaired DAergic metabolism (Collier et al., 2011, 2017), more specifically with a decrease in TH^+^ neurons, dopamine (DA) transporters, and the synthesis of DA during aging (Irwin et al., 1992; Rollo, 2009; Hindle, 2010; Collier et al., 2011). Inflammaging refers to the pro-inflammatory environment and the alterations in neuron–glia communication in aging tissue resulting from the functional decline of the immune system and characterized by an increase in pro-inflammatory markers (Franceschi et al., 2000). These factors and the chronic inflammatory state may contribute to the vulnerability of DAergic neurons (Migliore and Coppedè, 2009; Hindle, 2010; Collier et al., 2011) and promote PD in genetically or environmentally susceptible individuals (Franceschi et al., 2000; Franceschi and Campisi, 2014). Moreover, several studies point to chronic neuroinflammation as an event preceding and accompanying neuronal dysfunction (Franceschi and Campisi, 2014; Calabrese et al., 2018). However, what causes the degeneration of DAergic neurons in PD is still unknown and is likely a multifactorial etiology including genetic and environmental factors (Collier et al., 2017). There is limited characterization of the evolution of inflammaging and PD at the enteric level and few studies have compared the aging of the ENS and CNS in PD development. Moreover, exploring PD’s etiology in early-onset PD (EOPD, patients under 50) (Gasser, 2007), which has a poorer prognosis than late-onset PD, is crucial for understanding the disease’s early stages and the interplay of genetic and environmental factors (Schrag et al., 1998; Kalinderi et al., 2016).

Numerous studies have also reported gastrointestinal (GI) disturbances preceding the motor features of PD (Jost, 2003; Chaudhuri et al., 2006). The local nervous system of the GI tract, the enteric nervous system (ENS), regulates many local functions such as motility, nutrient detection and immune responses (Furness, 2012; Natale et al., 2021). These enteric neurons communicate with the mucosal immune system (Lakhan and Kirchgessner, 2010) and local inflammatory responses within the gut can influence ENS functions (Sharkey and Kroese, 2001). DAergic neurons constitute up to 20% of the upper GI neuronal population (Anlauf et al., 2003; Li et al., 2004) and several ENS neurons produce DA (Pfeiffer, 2011). Interestingly, PD patients have lower levels of GI DA (Singaram et al., 1995), suggesting that dysregulation of the DAergic system might be a factor underlying GI dysfunction in PD. The GI disorders could also originate from *α*-syn aggregates developing in the ENS (Wakabayashi et al., 1988; Goedert et al., 2013). Those elements underlie the implication of the gut-brain axis in PD and that the disease could result from an infection spreading first through the intestinal and olfactory mucosa, triggering the prodromal signs and symptoms of PD, as theorized by Dr. Braak (Braak et al., 2003a, 2003b). Considering the presence of ENS disorders prior to the onset of motor symptoms in PD, our central hypothesis was that age-related inflammation would affect neuronal function and promote the progression of PD, and that the inflammaging process would affect neuronal function in the ENS before the CNS, playing a key role in the progression of PD from the periphery to the brain. We tested these hypotheses by determining the impact of aging on neurodegeneration and inflammation, and whether age makes DAergic neurons more vulnerable to the neurotoxin MPTP in the mouse. We focused on markers of neurodegeneration (TH), oxidative stress (Nrf2) and inflammation (Iba1, GFAP) within the nigrostriatal pathway and the myenteric plexus, to compare the ENS and CNS sensitivity to MPTP in young (2–4 months) and adult (7–12 months) mice.

## 2 Materials and methods

### 2.1 Animals and MPTP injections

In this study, we used NF-κB^EGFP^ male mice with a C57BL6 background, aged from 2 to 12 months, obtained from Dr. Christian Jobin’s laboratory (FL, USA). The NF-κB knock-in mouse expresses enhanced green fluorescent protein (EGFP) under the transcriptional control of NF-κB cis elements (*cis*-NF-κB^EGFP^) (Magness et al., 2004). These transgenic mice were used to monitor NF-κB pathway activation, a central regulator of inflammation, in monocytes and macrophages. This model expresses EGFP under the control of NF-κB-responsive elements, allowing for the visualization and quantification of NF-κB activation. Previous studies (Côté et al., 2015) showed transient NF-κB activation post-MPTP in young mice, undetectable by day 5. This study aimed to assess whether NF-κB activation persists in aged mice at the same time point, potentially indicating prolonged inflammation. In our study, we solely utilized male mice, as female mice demonstrated resistance to MPTP at the administered dosage (Isenbrandt et al., 2021). All animals were housed and fed ad libitum at the Centre de recherche du CHU de Québec – Université Laval. All experiments were approved by the animal research committee of the Centre de recherche du CHU de Québec – Université Laval and performed according to the Canadian Guide for the Care and Use of Laboratory Animals (protocol number: 15–096). All efforts were made to minimize animal suffering and to reduce the number of mice used.

Twenty-four mice received four intraperitoneal (i.p.) injections of freshly prepared MPTP Hydrochloride (MPTP-HCl; 8 mg/kg free base; Sigma Aldrich Chemical, St. Louis, MO, USA) dissolved in saline 0.9%, in 2-h intervals. The control group comprised twenty mice that received four i.p. injections of a 0.9% saline solution at 2-h intervals. To avoid contamination, mice that received MPTP were housed separately from saline-treated control mice. Mice treated with saline and MPTP were separated into two age groups: young adults (2–4 months; *n* = 10 and *n* = 11 respectively) and adults (7–12 months; *n* = 10 and *n* = 13 respectively). In addition, the ages of the mice at the time of euthanasia were sufficiently distributed to allow us to conduct correlational studies between the age of death and the markers studied.

### 2.2 Tissue preparation

Animals were anesthetized with a ketamine and xylazine mixture (respectively, 100 mg/kg and 10 mg/kg) before intracardiac perfusion with 0.1 M phosphate-buffered saline (PBS). The brain and intestine were quickly removed and post-fixed with 4% paraformaldehyde (PFA) for 48 h, then dehydrated in a solution composed with 20% sucrose and 0.05% sodium azide in 0.1 M PBS pH 7.4 at 4 °C. Fixed brains were frozen in a dry ice and ethanol mixture, mounted on a microtome (Leica Microsystems Inc., ON, Canada), and cut into 30-μm-thick coronal sections. Collected brain sections were immersed in a tissue cryoprotectant solution (0.05 M PBS pH 7.3, 30% ethylene glycol and 20% glycerol) and stored at −20 °C until use for immunofluorescence. Five to six sections (1–10 mm^2^/section) of small intestine sampled in the distal ileum were micro-dissected to reveal the myenteric plexus hidden between muscle layers as described previously (Côté et al., 2011). Briefly, the submucosal layer and the circular muscle layer were separated from the longitudinal muscle layer under a stereomicroscope. Subsequently, four to six sections of small intestine were dissected to isolate the submucosal plexus by separating the mucosal layer from the underlying muscle under a stereomicroscope, as previously described (Ahrends et al., 2022). Each section was kept in PBS at 4 °C until use for immunohistochemistry or immunofluorescence.

### 2.3 Immunofluorescence analysis

Immunoreactivity quantifications and counts were performed by immunofluorescence. Myenteric plexus (five to six sections), submucosal plexus (four to six sections) and brain sections containing the striatum (four to five sections) or the SNpc (five to six sections) were incubated for 1 h at 100 °C in sodium citrate for antigen retrieval before a 30-min blocking treatment with a solution of 0.4% Triton X-100 and 5% donkey or goat serum (Sigma-Aldrich, Oakville, ON, Canada) in PBS 1X. Free-floating tissues were stained overnight with different primary antibodies, followed by a 2-h incubation with secondary antibodies conjugated to Alexa Fluor in blocking solution. Nuclear counterstaining with 0.022% DAPI (Invitrogen Corporation, Waltham, MA, USA) was performed before mounting sections. See Table 1 for antibody listing. Marked sections were imaged using a Zeiss AxioScan.Z1 Digital Slide Scanner and Zen 2.3 acquisition software (Carl Zeiss Canada). Micro-dissected myenteric and submucosal plexuses sections, as well as brain sections containing the SNpc were imaged per animal with a 20 × objective lens (NA 0.45). Brain sections of the striatum were imaged with a 10 × objective lens (NA 0.45). The contours of striatum (Bregma 0.86 mm), SNpc (Bregma −2.92 mm) and myenteric plexus of the ileum were traced with ImageJ Fiji software. Mean pixel intensity was determined on images for immunoreactivity quantification using MathWorks Matlab® 2018a software, and for density count, labeled cells were calculated as the number of positive cells per area (mm^2^) for each brain slice and section of ileum. Mean values were calculated with the microdissected sections for each animal (four to six sections per animal). All images were captured blindly, as were undertaken the data analyses (Côté et al., 2015).

**Table 1:** Primary and secondary antibodies.

Primary antibodies	Host	Company	Catalog #	Dilution
Tyrosine Hydroxylase	Rabbit	Pel-Freez Biologicals	P40101-150	1/1000
Tyrosine Hydroxylase	Sheep	Abcam	Ab113	1/200
Iba1	Rabbit	Wako	019–19,741	1/250
GFAP	Mouse	Millipore	MAB360	1/1000
Nrf2	Rabbit	Abcam	Ab62352	1/100

Secondary antibodies	Host	Company	Catalog #	Dilution
Anti-Rabbit Biotinylated	Goat	Vector Laboratories	BA1000	1/1000
Anti-Sheep Cy3	Donkey	Millipore	AP184C	1/1000
Anti-Rabbit IgG Alexa FluorTM 488	Donkey	Life Technologies	A21206	1/1000
Anti-Mouse IgG Alexa FluorTM 488	Goat	Abcam	Ab150113	1/1000
Anti-Rabbit AlexaFluor 555	Donkey	Life Technologies	A31572	1/1000

### 2.4 Immunohistochemistry analysis

For visual detection of myenteric neurons, five to six sections of myenteric plexus were incubated for 60 min at 37 °C in the Cuprolinic Blue (Polysciences Inc., PA, USA) (Holst and Powley, 1995) to stain neurons. Free-floating myenteric plexus sections were treated for 30 min with a blocking solution containing 0.4% Triton X-100, 4% goat serum and 1% bovine serum albumin (Sigma-Aldrich) in PBS. Sections were immunostained overnight in the previous solution with a rabbit polyclonal anti-TH (1:1000, Pel-Freez Biologicals, AR, USA) to stain DAergic neurons. Sections were washed and then incubated with secondary antibody goat anti-rabbit biotinylated (1:250, Chemicon International, MA, USA) for 2 h. Staining was visualized with the ABC amplification system (Vector Laboratories Inc., ON, Canada) and the 3,3’diaminobenzidine (DAB, Vector Laboratories Inc.) according to manufacturer guidelines. Myenteric plexus sections were imaged using a Zeiss AxioScan. Z1 Digital Slide Scanner and Zen 2.3 acquisition software (Carl Zeiss Canada) with a 20 × objective lens (NA 0.45). Five to six microdissected myenteric plexus sections and brain sections of SNpc were imaged per animal with three images per sections. The investigator, blinded to treatment groups, section contours were drawn as a virtual overlay on images. For TH^+^ and myenteric neurons density count, we performed a systematic quantification of all labeled cells in the structure per image stack, by counting the number of positive cells per area in squared millimeters, using NIH Fiji software (ImageJ 1.54f Java 1.8.0_322). Subsequently, TH^+^ submucosal neurons were similarly quantified using Zen imaging software (Carl Zeiss, version 3.8.1). Mean values were calculated with the microdissected sections for each animal (Côté et al., 2015).

### 2.5 Statistical analysis

Data were evaluated with the Shapiro–Wilk test to determine normality; outliers were discarded using the interquartile range method. Comparisons between groups were calculated by two-way analysis of variance (ANOVA) followed by Tukey’s *post-hoc* tests, with MPTP treatment and age as independent variables. Results are represented as the mean ± SEM of 6–12 mice per group. For the correlation analysis, Pearson correlation coefficient was calculated, and simple linear regression was performed. Results are considered statistically significant if *p* < 0.05. All statistical analyses were performed with Prism 9 (GraphPad Software Inc., La Jolla, CA, USA) software.

## 3 Results

### 3.1 Effects of aging and MPTP on nigrostriatal neurodegeneration

**Figure 1:**
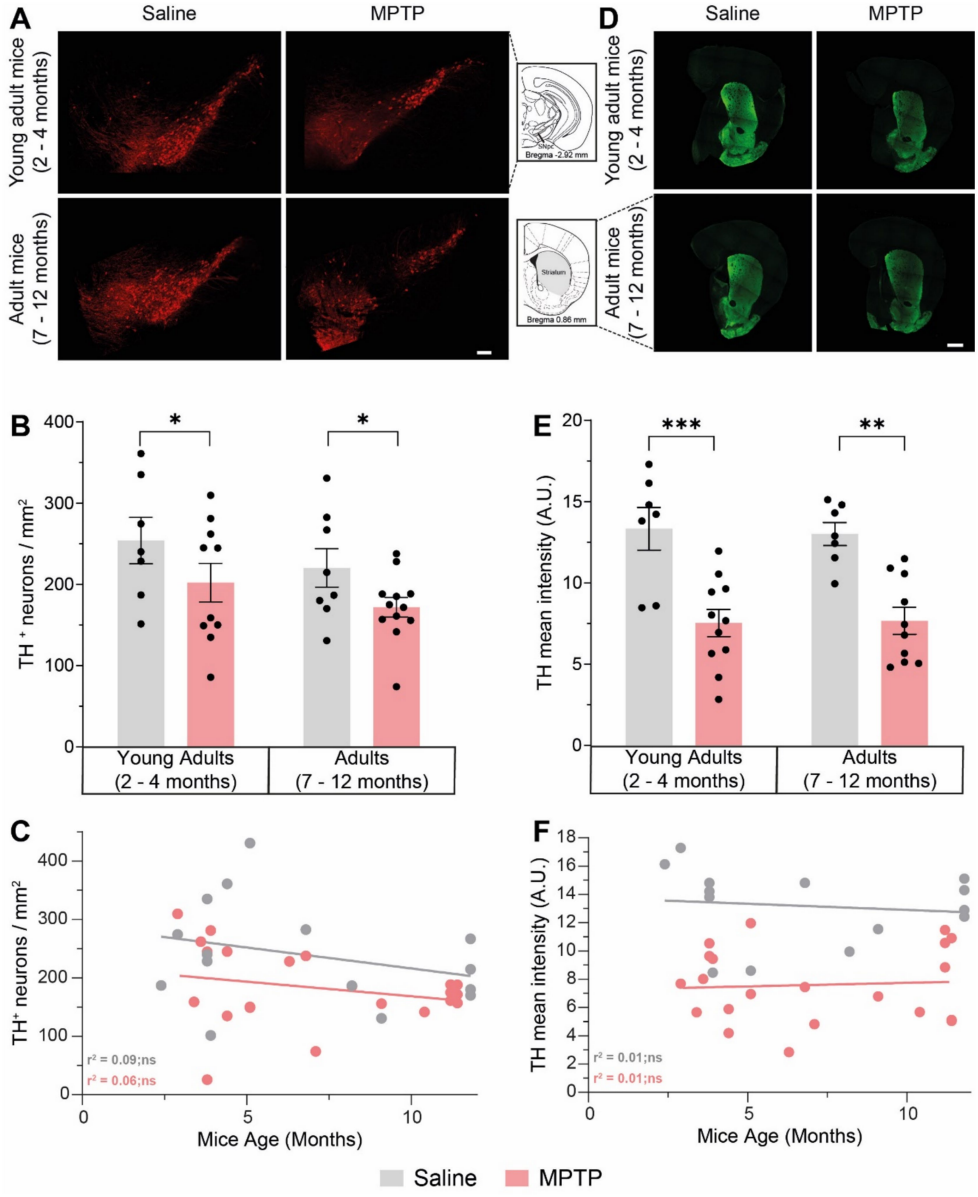
Effect of age and MPTP treatment on TH + neurons in the substantia nigra pars compacta (SNpc) and on the TH + fibers in the striatum. **(A)** Representative microphotographs of TH staining in the SNpc. Scale bar = 100 μm. **(B)** TH^+^ neuronal population density in the SNpc of saline and MPTP treated mice. Values shown are the mean cell count /mm^**2**^ tissue area ± SEM (7–12 mice per group). **(C)** Correlation between TH^+^ neuronal population density in the SNpc and the age of saline and MPTP treated mice. Values shown are the individual cell counts for each mouse, the linear regression and the *r*^2^ values. **(D)** Representative microphotographs of TH staining in the striatum. Scale bar = 500 μm. **(E)** TH mean intensity in the striatum of saline and MPTP treated mice. Values shown are the mean intensity in tissue area ± SEM (7–12 mice per group). **(F)** Correlation between TH mean intensity in the striatum and the age of saline and MPTP treated mice. Values shown are the individual measures for each mouse, the linear regression and the *r*^2^ values. *p < 0.05, **p < 0.01, ***p < 0.001. 2-way ANOVA, Tukey test for **B** and **E**. Pearson correlation coefficient for **C** and **F**.

The loss of nigrostriatal DAergic neurons is a hallmark of neuropathological changes in the brains of PD patients and can be observed in most animal models (Damier et al., 1999; Cannon and Greenamyre, 2010; Cheng et al., 2010; Coulombe et al., 2018; Isenbrandt et al., 2021; Lamontagne-Proulx et al., 2023). Hence, we investigated the integrity of the nigrostriatal pathway after MPTP injections and during aging using TH, a rate-limiting enzyme in the synthesis of DA, as a marker for DAergic neurons and their projections. Quantification of DAergic neuron density in SNpc revealed a 20.5% loss of nigral neurons in young adult mice treated with MPTP (*p* = 0.0263; Figures 1A,B) and a 21.8% loss in adult mice treated with MPTP (p = 0.026). There was no significant difference in DAergic neurons density between young and older mice, either treated with saline or MPTP (respectively *p* = 0.7429 and *p* = 0.6931). There was no correlation between TH^+^ neuron density in the SNpc and age in the saline (r^2^ = 0.09; *p* = 0.2865; Figure 1C) and MPTP group (*r*^2^ = 0.06; *p* = 0.2678).

TH labeling for DAergic projections in the striatum showed a 43.5% loss of in young adults treated with MPTP (*p* = 0.0007; Figures 1D,E) and a 41% loss in adult mice treated with MPTP (*p* = 0.0022). There was no significant difference in mean TH intensity between young and adult mice, either treated with saline or MPTP (respectively *p* = 0.9960 and *p* = 0.9994). There was no correlation between TH intensity in the striatum and age in the saline (*r*^2^ = 0.01; *p* = 0.6827; Figure 1F) and MPTP group (*r*^2^ = 0.01; *p* = 0.7830). Therefore, our results indicate that MPTP causes DAergic neurodegeneration in the nigrostriatal pathway of both young and adult mice, but aging does not impact DAergic neuronal density in the SNpc and DAergic projections in the striatum.

**Figure 2:**
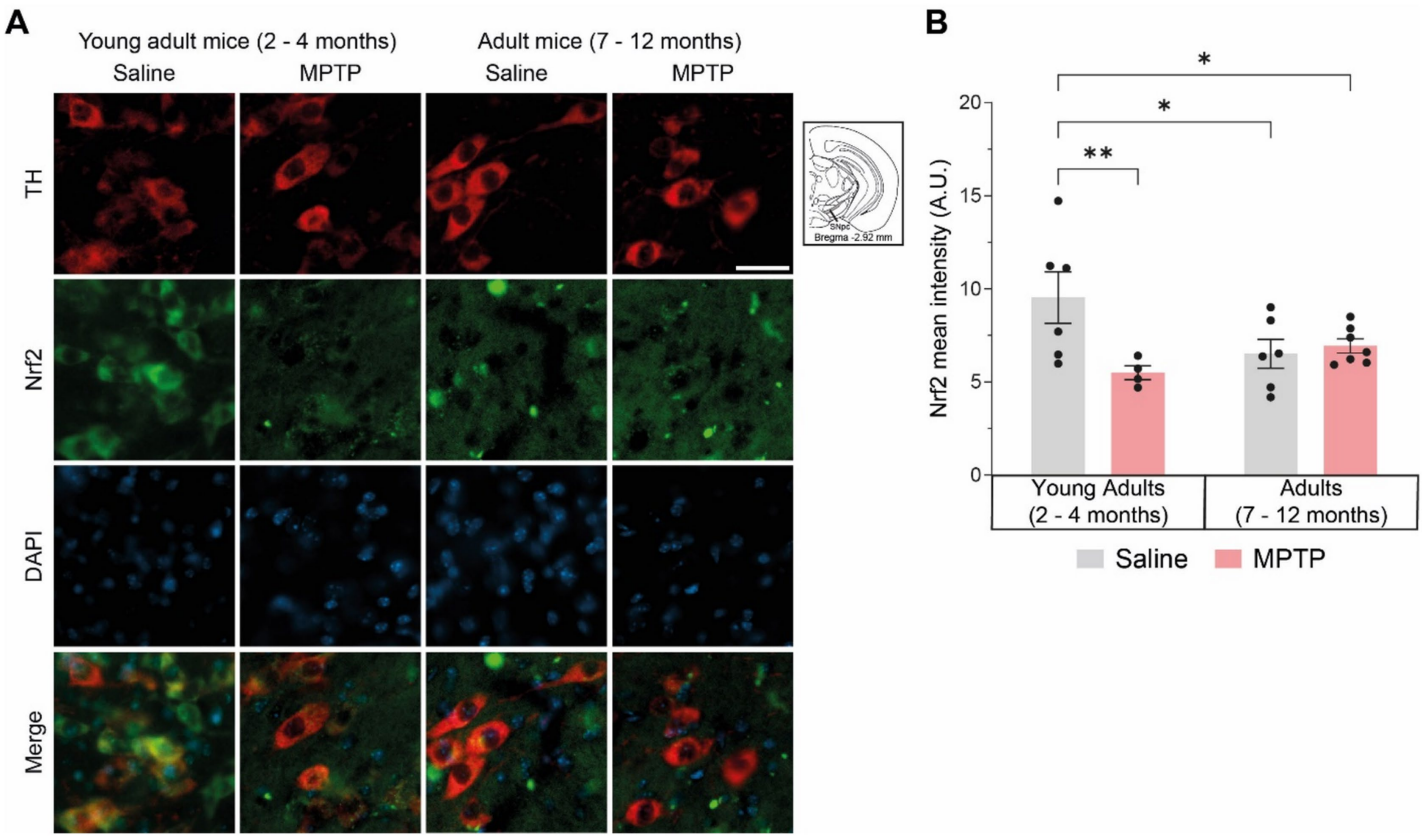
Effect of age and MPTP treatment on oxidative stress in the substantia nigra pars compacta (SNpc). **(A)** Photomicrographs of TH (red) and Nrf2 (green) immunofluorescence show colocalization of TH enzyme and Nrf2 protein in the substantia nigra pars compacta (SNpc) of mice. Scale bar = 20 μm. **(B)** Immunoreactivity quantification of Nrf2 colocalizing with TH (dopaminergic neuron body). Values shown are the mean intensity in tissue area ± SEM (4–10 mice per group). *p < 0.05, **p < 0.01. 2-way ANOVA, Tukey test.

Oxidative stress is an important pathological feature detected in PD patients and altered gene expression regulating antioxidative responses could lead to cell death (Nikam et al., 2009). To assess the ability of nigral DAergic neurons to defend themselves against oxidative stress, we used immunofluorescence to quantify the expression of the transcription factor Nrf2 [nuclear factor (erythroid-derived 2)-like 2] protein inside the neuron cytoplasm (Figure 2A). Results showed that young adult mice treated with MPTP had a lower level of cytoplasmic Nrf2 protein in DAergic neuron bodies compared with young animals treated with saline (*p* = 0.0076) (Figure 2B). Additionally, older mice treated with either saline or MPTP presented a lower level of the transcription factor compared with young saline-treated mice (respectively *p* = 0.0219 and *p* = 0.0379). MPTP treatment in adult mice did not alter the levels of Nrf2 compared to saline treatment (*p* = 0.7232). These findings suggest that MPTP as well as physiological aging can decrease Nrf2 protein levels and thereby reduce the antioxidant response.

### 3.2 Effects of aging and MPTP on nigrostriatal inflammation

**Figure 3:**
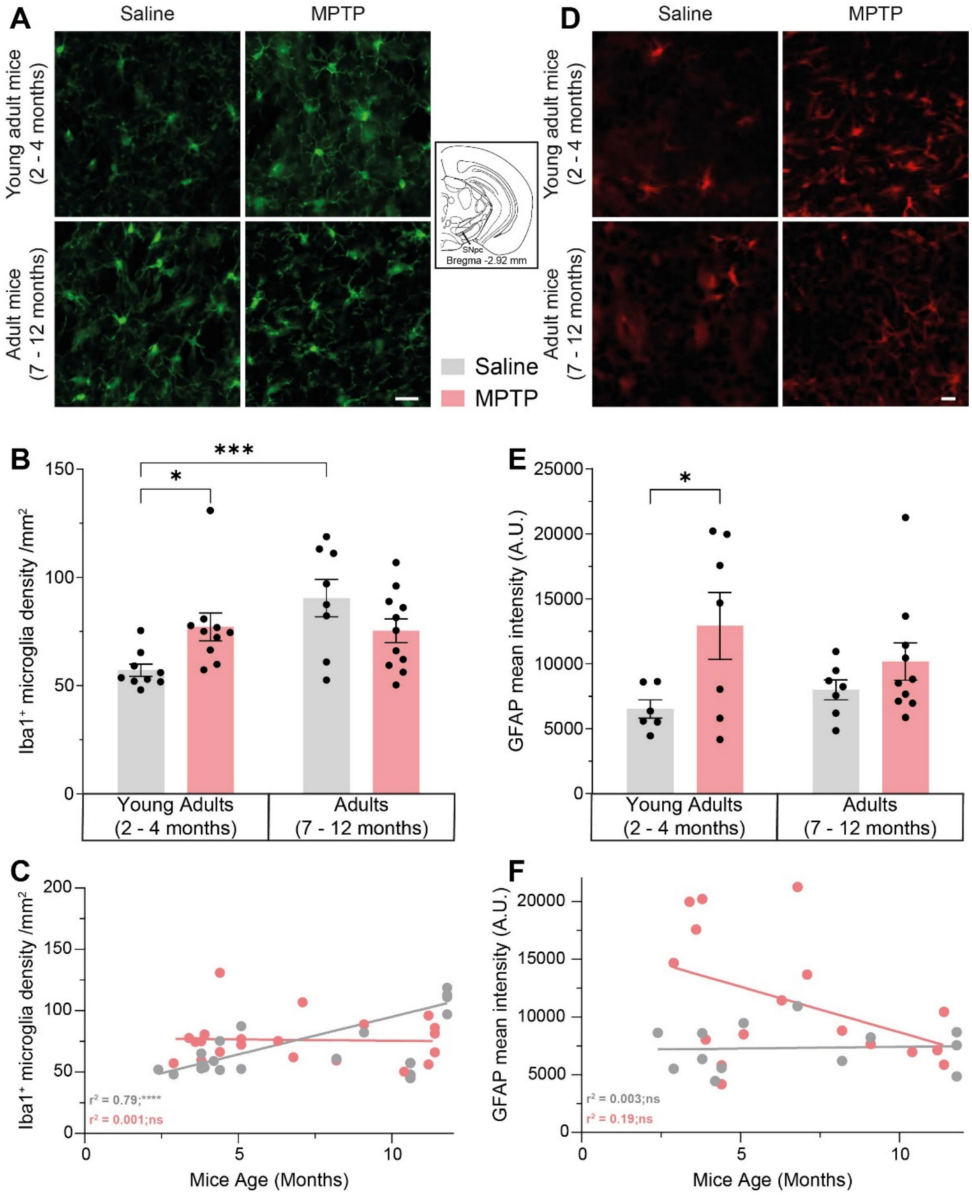
Effect of age and MPTP treatment on microgliosis and astrogliosis in the substantia nigra pars compacta (SNpc). **(A)** Representative microphotographs of Iba1 staining in the SNpc. Scale bar = 20 μm. **(B)** Iba1^+^ cells density in the SNpc of saline and MPTP treated mice. Values shown are the mean cell count /mm^**2**^ tissue area ± SEM (8–11 mice per group). **(C)** Correlation between Iba1^+^ neuronal population density in the SNpc and the age of saline and MPTP treated mice. Values shown are the individual cell counts for each mouse, the linear regression and the r^2^ values. **(D)** Representative microphotographs of GFAP staining in the SNpc. Scale bar = 20 μm. **(E)** GFAP mean intensity in the SNpc of saline and MPTP treated mice. Values shown are the mean intensity tissue area ± SEM (7–10 mice per group). **(F)** Correlation between GFAP mean intensity in the SNpc and the age of saline and MPTP treated mice. Values shown are the individual measures for each mouse, the linear regression and the r^2^ values. *p < 0.05, ***p < 0.001, ****p < 0.0001. 2-way ANOVA, Tukey test for **B** and **E**. Pearson correlation coefficient for **C** and **F**.

Multiple lines of evidence suggest that the activation of the immune system could play an essential role in neurodegeneration (Guzman-Martinez et al., 2019). Our analysis of microgliosis through the density of the ionized calcium-binding adapter molecule Iba1^+^ cells in the SNpc showed a 35% increase in young adult mice with MPTP (*p* = 0.0250; Figures 3A,B), as well as a 58.2% increase with aging in saline-treated mice (*p* = 0.0008). There was a correlation between microglia density and age in saline mice (*r*^2^ = 0.79; *p* < 0.0001; Figure 3C), but not in mice receiving MPTP (*r*^2^ = 0.001; *p* = 0.8726). We also observed astrogliosis through the immunoreactivity of the glial fibrillary acidic protein (GFAP) in the SNpc of young adult mice exposed to MPTP, with an increase of 98% (*p* = 0.0148; Figures 3D,E), but not in adult mice (*p* = 0.3241). There was no correlation between GFAP intensity and age either in the saline group (*r*^2^ = 0.003; *p* = 0.8635; Figure 3F) or MPTP group (*r*^2^ = 0.19; *p* = 0.0804). Those results show that MPTP induces microgliosis and astrogliosis in young mice SNpc and that aging causes microgliosis in saline-treated mice, but also alters the inflammatory response of mice to MPTP.

**Figure 4:**
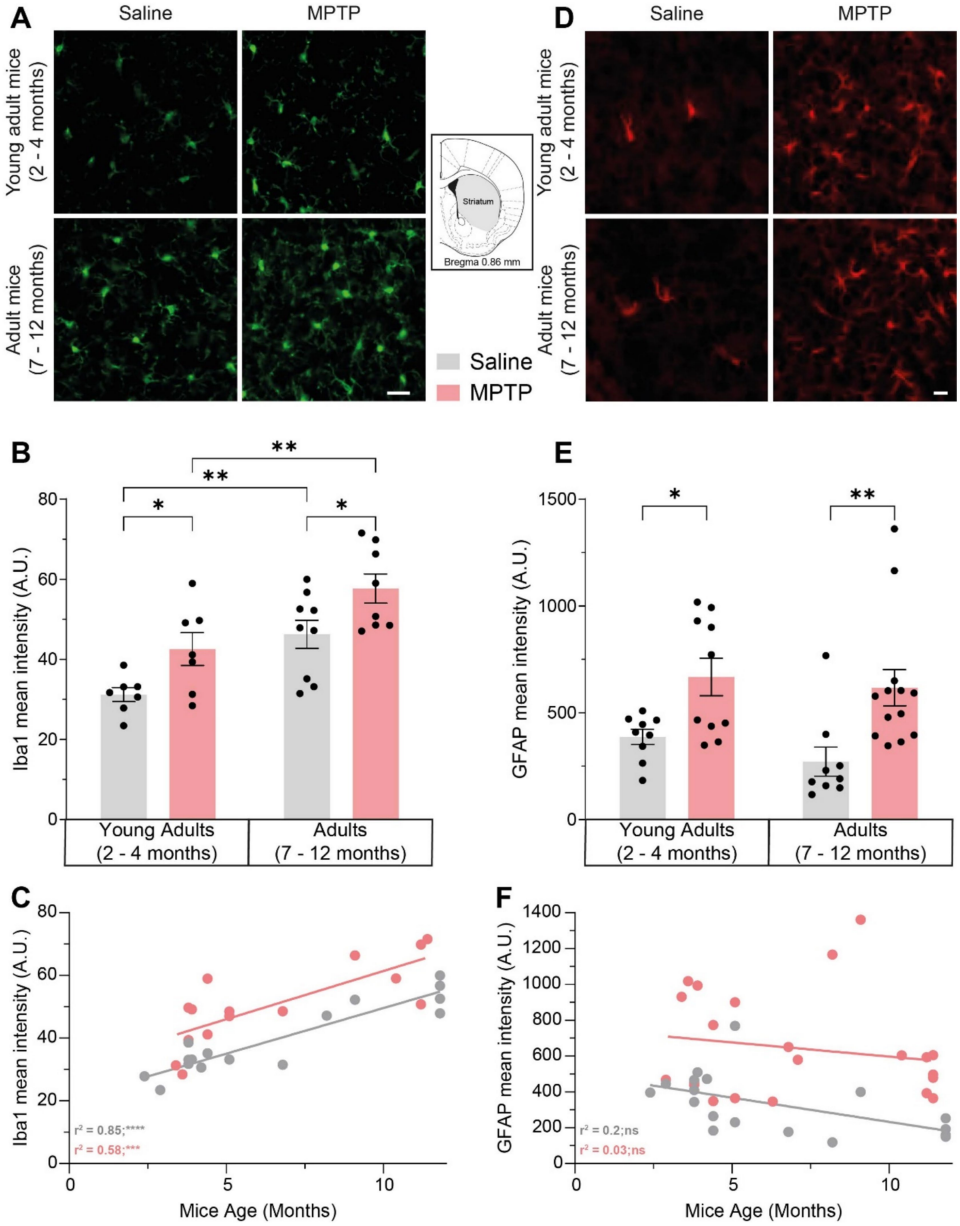
Effect of age and MPTP treatment on microgliosis and astrogliosis in the striatum. **(A)** Representative microphotographs of Iba1 staining in the striatum. Scale bar = 20 μm. **(B)** Iba1 mean intensity in the striatum of saline and MPTP treated mice. Values shown are the mean intensity in tissue area ± SEM (7–9 mice per group). **(C)** Correlation between Iba1 mean intensity in the striatum and the age of saline and MPTP treated mice. Values shown are the individual measures for each mouse, the linear regression and the r^2^ values. **(D)** Representative microphotographs of GFAP staining in the striatum. Scale bar = 20 μm. **(E)** GFAP mean intensity in the striatum of saline and MPTP treated mice. Values shown are the mean intensity in tissue area ± SEM (9–13 mice per group). **(F)** Correlation between GFAP mean intensity in the striatum and the age of saline and MPTP treated mice. Values shown are the individual measures for each mouse, the linear regression and the *r*^2^ values. *p < 0.05, **p < 0.01, ***p < 0.001, ****p < 0.0001. 2-way ANOVA, Tukey test for **B** and **E**. Pearson correlation coefficient for **C** and **F**.

MPTP injections induced a 36.5% Iba1 increase in the striatum of young adult mice (*p* = 0.0348) and a 24.6% increase in adult mice (*p* = 0.0207). We also observed a 48.4% increase of microgliosis with aging in the striatum of saline-treated mice (*p* = 0.0043; Figures 4A,B) as well as a 35.4% increase in MPTP-treated mice (*p* = 0.0051). However, there was a stronger correlation between Iba1 levels and age in saline-treated mice (*r*^2^ = 0.85; *p* < 0.0001; Figure 4C) than in MPTP-treated mice (*r*^2^ = 0.58; *p* = 0.0009). Remarkably, the level of microgliosis due to aging was similar to the MPTP-dependent microgliosis observed in young mice. Similar results were observed regarding the impact of MPTP on astrogliosis in the striatum, inducing a 72.5% increase of GFAP levels in young adult mice (*p* = 0.0178; Figures 4D,E) and a 127% increase in older mice (*p* = 0.0025). There was no correlation between GFAP intensity and age either in the saline group (*r*^2^ = 0.2; *p* = 0.0687; Figure 4F) or MPTP group (*r*^2^ = 0.03; *p* = 0.4107). Those analyses indicated an increase in microgliosis with aging, but not astrogliosis. MPTP induced an increase in pro-inflammatory response in the striatum of young and older mice, but only in the SNpc of young mice. This indicates that MPTP causes microgliosis and astrogliosis in the striatum of both young and older mice, however, only microgliosis is observed with aging.

### 3.3 Effects of aging and MPTP on enteric neurodegeneration

**Figure 5:**
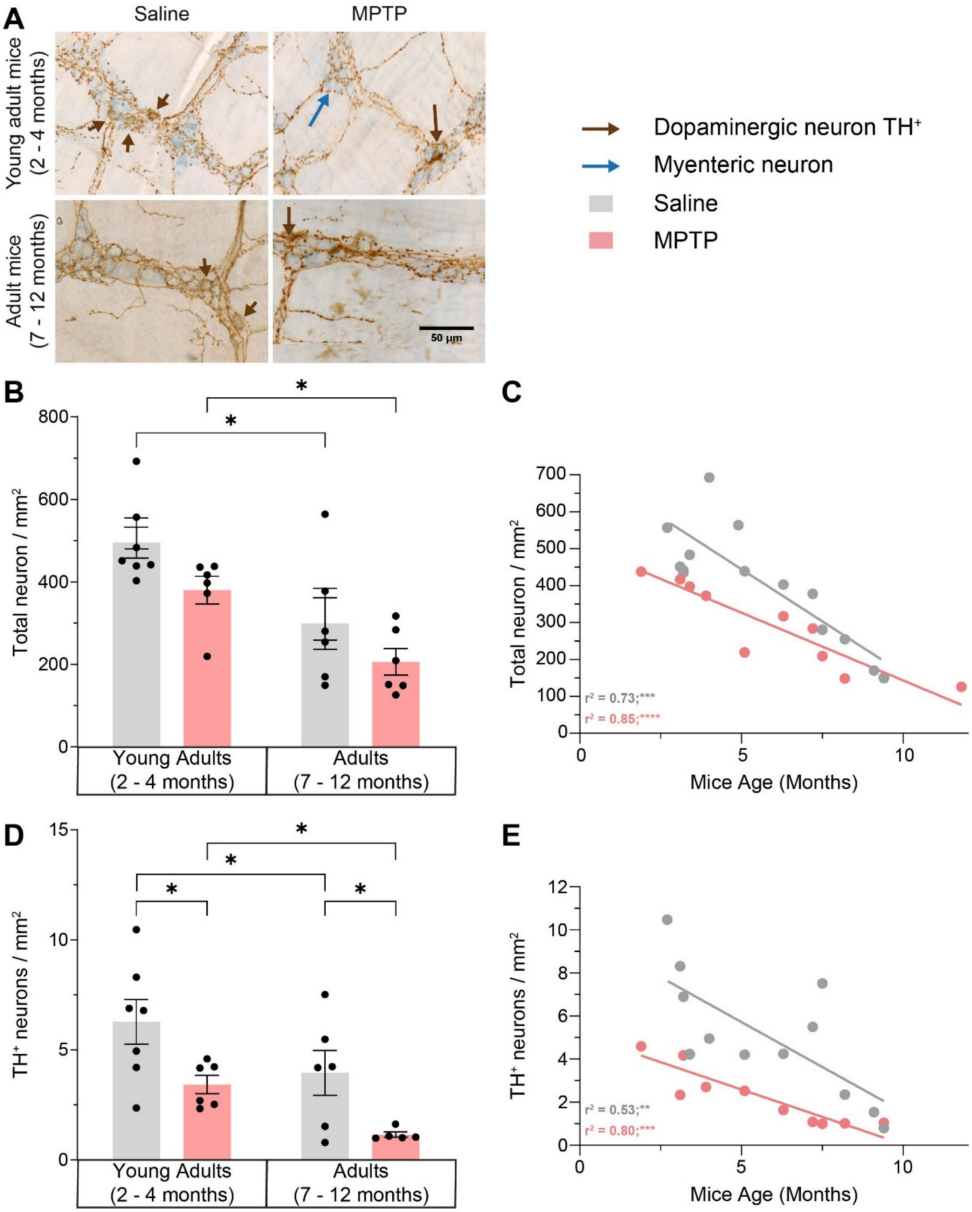
Effect of age and MPTP treatment on total neuronal population and TH + neurons in the myenteric plexus. **(A)** Representative microphotographs of neuronal (in blue) and TH (in brown) staining. Scale bar = 50 μm. **(B)** Total neuronal population density in the myenteric plexus of saline and MPTP treated mice. Values shown are the mean cell count /mm^**2**^ tissue area ± SEM (6–7 mice per group). **(C)** Correlation between total neuronal population density in the myenteric plexus and the age of saline and MPTP treated mice. Values shown are the individual cell counts for each mouse, the linear regression and the r^2^ values. **(D)** TH^+^ neuronal population density in the myenteric plexus of saline and MPTP treated mice. Values shown are the mean cell count /mm^2^ tissue area ± SEM (6–7 mice per group). **(E)** Correlation between TH^+^ neuronal population density in the myenteric plexus and the age of saline and MPTP treated mice. Values shown are the individual cell counts for each mouse, the linear regression and the *r*^2^ values. *p < 0.05, **p < 0.01, *** *p* < 0.001, ****p < 0.0001. 2-way ANOVA, Tukey test for **B** and **D**. Pearson correlation coefficient for **C** and **E**.

We next examined the impact of aging on the global neuronal population and MPTP-induced DAergic neuronal alterations in the myenteric plexus of the GI tract. We observed a 39.4% decrease in the number of total neurons in the myenteric plexus of the ileum in saline-treated mice (*p* = 0.0174; Figures 5A–C) and a 45.8% decrease in MPTP-treated mice (*p* = 0.0482) strongly correlated with age (respectively r^2^ = 0.73; *p* = 0.0002 and r^2^ = 0.85; p < 0.0001). The exposure to MPTP did not affect total neuronal density in the myenteric plexus. A similar decrease in DAergic neurons was observed with aging. In mice treated with saline, there was a 37% loss of DAergic neurons with age (*p* = 0.0497; Figures 5A,D,E) and a 66.5% decrease in mice treated with MPTP (*p* = 0.0112). This DAergic enteric neurodegeneration was correlated with age (*r*^2^ = 0.53; *p* = 0.0075 in saline mice and *r*^2^ = 0.80; *p* = 0.0002 in MPTP mice). In young adult mice, MPTP treatment induced a 45.4% decrease in the density of DAergic neurons (*p* = 0.0187) and a 71% loss in older mice (*p* = 0.0311). Interestingly, this enteric neurodegeneration was stronger in adult mice injected with MPTP. Remarkably, the level of DAergic neurodegeneration due to aging in the myenteric plexus was similar to the MPTP-dependent DAergic loss observed in young mice. Thus, our results suggest that MPTP as well as aging result in an important loss of enteric DAergic neurons, however, this loss is aggravated in MPTP adult mice.

Thereafter, the impact of aging on the global population and MPTP-induced DAergic neuronal alterations in the submucosal plexus of the GI tract was assessed. In young mice, TH^+^ neuronal density was comparable between saline-treated and MPTP-treated animals (13.308 ± 1.210 neurons/mm^2^ vs. 14.321 ± 1.929 neurons/mm^2^; *p* = 0.6595) (Supplementary Figure 1). Similarly, in adult mice, MPTP treatment did not significantly alter TH^+^ neuronal density (16.242 ± 1.816 neurons/mm^2^ in saline vs. 15.146 ± 1.039 neurons/mm^2^ in MPTP-treated mice; *p* = 0.6055). No significant age-related decreased was detected between young and adult groups in either treatment condition (*p* = 0.2190 for saline; *p* = 0.6873 for MPTP). Thus, our data suggest that neither MPTP nor aging result in an important loss of submucosal DAergic neurons.

### 3.4 Effects of aging and MPTP on enteric inflammation

**Figure 6:**
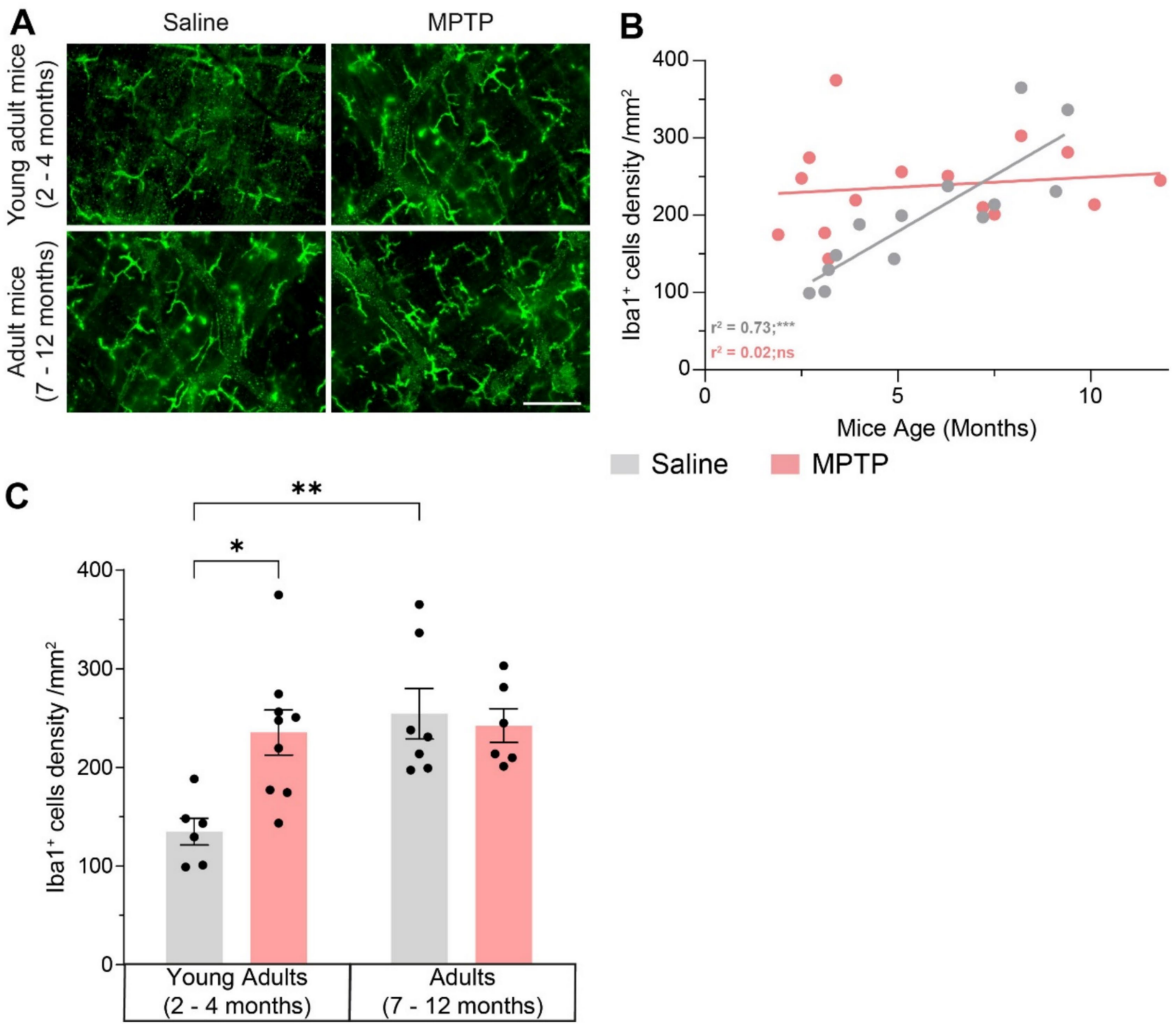
Effect of age and MPTP treatment on macrophages in the myenteric plexus. **(A)** Representative microphotographs of Iba1 staining. Scale bar = 100 μm. **(B)** Correlation between Iba1^+^ macrophages density in the myenteric plexus and the age of saline and MPTP treated mice. Values shown are the individual cell counts for each mouse, the linear regression and the *r*^2^ values. **(C)** Iba1^+^ macrophages density in the myenteric plexus of saline and MPTP treated mice. Values shown are the mean cell count /mm^2^ tissue area ± SEM (6–8 mice per group). *p < 0.05, **p < 0.01, ***p < 0.001. Pearson correlation coefficient for **B**. 2-way ANOVA, Tukey test for **C**.

To explore inflammation at the myenteric level, we analyzed the density of total macrophages Iba1^+^ (Figures 6A–C). We observed a 74% increase of Iba1^+^ macrophages density in young adult mice treated with MPTP compared to young mice treated with saline (*p* = 0.0145), but not in adult mice treated with MPTP compared to saline adult mice. We also observed an 88% rise in macrophages density with age in saline-treated mice (*p* = 0.0054), which is comparable to the enteric inflammation caused by MPTP. This rise was strongly correlated with age (*r*^2^ = 0.73; *p* = 0.0002). These results suggest that aging causes enteric inflammation and that MPTP induces a similar enteric inflammation only in young mice, but no additional inflammation in older mice.

## 4 Discussion

While age is the main risk factor of PD, how it impacts the etiopathogenesis and progression of the disease remains poorly understood. The ENS is important in the context of PD as GI disorders often precede motor symptoms and are reported by most PD patients (Natale et al., 2008; Poirier et al., 2016). As such, we hypothesized that DAergic neurons were affected earlier in the ENS than in the CNS, with the inflammaging process affecting the ENS before the CNS. To understand early aging’s role in PD, akin to its prodromic phase, we studied adult mice rather than elderly ones. This study used a mild MPTP regimen to simulate early PD stages. We began by evaluating the neural densities to validate our model, and we found that MPTP caused selective damages to striatal DAergic terminals (41–43% decrease in the density of TH^+^ projections) with limited loss of DAergic cells in the SNpc (21% of neuronal loss). This was accompanied by a reduction of the antioxidant capacity in the DAergic neurons of the SNpc. Acute MPTP treatment in the present study also induced astroglial activation and microgliosis in the nigrostriatal pathway, which is similar to results obtained in other studies performed with both acute and chronic MPTP protocols and data on neuroinflammation in PD patients (McGeer and McGeer, 2008; Hirsch and Hunot, 2009). It also prompted macrophages infiltration in the myenteric plexus. Those results are supported by previous studies using a similar model (Côté et al., 2011, 2015; Bourque et al., 2013; Dou et al., 2018; Jarras et al., 2020), highlighting MPTP’s suitability for studying peripheral inflammation and inflammaging in PD pathogenesis (Devos et al., 2013; Calabrese et al., 2018), despite its inability to mimic its slow progression. However, despite increased macrophage density, no GFP signal was detected in young or aged mice 5 days after MPTP injections (data not shown), suggesting that NF-κB activation is not detectable at this stage, indicating a low-grade inflammation. In the present study, MPTP induced a 39% loss of DAergic neurons in the myenteric plexus of young and adult mice, without affecting other enteric neurons, confirming MPTP’s neurotoxic specificity. Moreover, the absence of global neuronal damage, as measured by the total neuron population, aligns with the study of Natale *et al.* reporting that the loss of TH^+^ cells in the gut is selectively due to the disappearance of DAergic neurons (Anderson et al., 2007; Natale et al., 2010). However, other studies have reported no loss of enteric DAergic neurons in patients (Lebouvier et al., 2009, 2010; Annerino et al., 2012). The present data support that alterations in both central and peripheral TH^+^ neurons may be features of PD, consistent with some studies in patients (Singaram et al., 1995; Lebouvier et al., 2008).

The ENS comprises a highly diverse population of neurons and neurotransmitters. Those neurons, constituting less than 1% of all enteric cells, are organized into ganglionic plexuses—primarily the myenteric (MyP) and submucosal (SMP) plexuses—or into non-ganglionic nerve fiber bundles (Furness and Costa, 1980; Brehmer, 2021). Functionally, enteric neurons are typically classified into five major categories based on their neurotransmitter profiles: (1) excitatory motor neurons (cholinergic), (2) inhibitory motor neurons (nitrergic), (3) sensory neurons, (4) excitatory and inhibitory interneurons, and (5) secretomotor/vasodilator neurons (Natale et al., 2021). The cholinergic neurons make up the majority, at least 70% of myenteric neurons (Anlauf et al., 2003). DAergic neurons represent a relatively small but functionally significant subset of the ENS. In mice, they account for approximately 10–13% of enteric neurons, while in humans, this proportion can reach up to 20%, with a distribution that follows an oral-to-aboral gradient (Anlauf et al., 2003; Li et al., 2004). These neurons are particularly enriched in the upper gastrointestinal tract, where they constitute 14–20% of the neuronal population, but their prevalence drops to 1–6% in the distal small intestine. DAergic enteric neurons express key dopaminergic markers, including tyrosine hydroxylase (TH), dopamine transporter (DAT), and all five dopamine receptor subtypes (D1–D5). Although their precise physiological role remains incompletely understood, evidence suggests that they exert an inhibitory influence on intestinal motility (Lebouvier et al., 2009). Expression of the dopaminergic phenotype in the mouse ENS is variable depending on the region of the gut, TH transcripts being more prominent in the stomach and ileum compared to the colon (Chalazonitis et al., 2022). The present results show that MPTP enhances neurotoxicity in enteric DAergic neurons of older mice, correlating negatively with age and neuron count in the plexus. This heightened sensitivity of enteric neurons to MPTP in older mice may be attributed to factors such as peripheral aged-related inflammation, reduced antioxidant capacity, increased oxidative stress, and decreased neurogenesis with age. Previous studies support these data, C57BL/6 mice receiving MPTP exhibiting dose-dependent decreases in DA levels in the striatum and SN (Sugama et al., 2003). Age-related susceptibility to MPTP may involve factors such as blood–brain barrier (BBB) penetration and MPP^+^ conversion rates. Monoamine oxidase B (MAO-B) levels increase with age and are doubled in the SN of PD patients (Damier et al., 1996), and show increased activity in the striatum of mice aged 2 to 10 months (Irwin et al., 1992). This may indicate that age enhances MPTP neurotoxicity, with older mice showing greater degeneration and inflammation in enteric DAergic neurons and the striatum specifically.

Despite the lack of astrogliosis in the SNpc and striatum of saline-treated older mice, the activation of microglia and macrophages differs between young and older mice, MPTP producing greater striatal inflammation in aged mice than in younger ones. MPTP also caused inflammation mainly in younger mice’s SNpc, while older mice displayed less sensitivity in the SNpc compared to the striatum. The striatum is more sensitive to MPTP toxicity, due to differences in DA transporter expression and metabolic activity (Smeyne et al., 2016; Lu et al., 2018; Ayerra et al., 2024). Aging also influenced inflammation in the myenteric plexus, with MPTP exposure causing inflammation in young mice but not exacerbating it in older ones. This suggests differential activation of microglia in the SNpc and macrophages in the gut between young and older mice, with persistent microglial activation contributing to increased toxicity. The absence of MPTP-related inflammation in aged mice could also result from a delayed neuroinflammatory response, as the aging immune system is less efficient, leading to slower macrophage activity and T lymphocyte reactions and being less efficient in distinguishing between endogenous and exogenous substances (Shaw et al., 2013; Heavener and Bradshaw, 2022). Other studies using a chronic MPTP model have shown less cytokine production and a delayed inflammatory response in the SN of older mice compared to young mice (Muñoz-Manchado et al., 2016; Yao and Zhao, 2018, p. 124). Inflammation might therefore occur later or with prolonged MPTP exposure due to the aged immune system’s reduced responsiveness.

DAergic neurons in the ENS share molecular features with midbrain dopaminergic neurons, expressing common transcription factors critical for dopaminergic identity and maintenance (Chalazonitis et al., 2020, 2022). Both systems utilize dopamine as a key neurotransmitter, although their targets and functions differ. SNpc DAergic neurons regulate critical brain functions such as motor control, motivation, and working memory (Chinta and Andersen, 2005; Chalazonitis et al., 2022). In contrast, enteric DAergic neurons influence gastrointestinal (GI) motility and exhibit regional variability in phenotype along the gut. These neurons often co-express dopamine with other neurotransmitters, contributing to a complex neurochemical identity (Li et al., 2004). Despite sharing dopaminergic identity, neurons in the ENS and the SNpc exhibit distinct developmental, anatomical, and functional features. Developmentally, DAergic neurons in the CNS emerge earlier than those in the ENS. These populations arise from different embryological origins, although they share common growth factors and signaling pathways during development (Chalazonitis et al., 2022). Electrophysiologically, both neuronal populations are characterized by sustained excitability and firing patterns. This persistent activity is thought to increase metabolic stress, making them more susceptible to degeneration, especially with aging (Lerner et al., 2015; Chalazonitis et al., 2022). However, the microenvironment in which these neurons operate differs significantly. SNpc neurons are protected by the blood–brain barrier (BBB), whereas enteric neurons—particularly those in the submucosal plexus—lack such protection. This exposes them directly to luminal contents and the gut microbiota, increasing their susceptibility to environmental insults and inflammatory stressors (Chalazonitis et al., 2022). Both SNpc and ENS DAergic neurons are implicated in Parkinson’s disease. In PD, *α*-synuclein aggregates have been found in both the substantia nigra and the submucosal plexus of the gut, suggesting a shared vulnerability and possibly a gut-to-brain propagation of pathology (Chalazonitis and Rao, 2018; Brudek, 2019; Chalazonitis et al., 2022). The present findings reveal that basal microgliosis occurs in the SNpc and striatum with aging, as well as reduced antioxidant response in the SNpc, while TH levels remained unaltered. However, aging in mice led to a decrease in the total neuronal population and a loss of DAergic neurons in the myenteric plexus. Other studies observed damage to the ENS with aging, with alterations in the size, shape, and distribution of myenteric neurons (Gomes et al., 1997; Hanani et al., 2004; Saffrey, 2013). Although age-related loss of myenteric neurons, particularly cholinergic neurons, is widely reported, data on extensive neuronal death in the ENS during aging are variable (Phillips and Powley, 2001; Phillips et al., 2003; Thrasivoulou et al., 2006; Bernard et al., 2009; Saffrey, 2013). Natale et al. (2010) conducted their analyses by comparing neuronal loss in the duodenum (a segment of the small intestine) and the colon, reporting a pronounced reduction in both submucosal and myenteric plexus TH^+^ neurons. This contrasts in part with our findings. In our study, we did not observe a significant change in the density of TH^+^ neurons in the submucosal plexus in the ileum, regardless of age. In this region, only TH^+^ neurons within the myenteric plexus appeared sensitive to MPTP-induced neurotoxicity. It is important to note that the regions of the small intestine analyzed differ between the two studies—duodenum in Natale et al. (2010) versus ileum in the present study—suggesting that enteric neuron vulnerability to MPTP may vary across different intestinal segments. Furthermore, the MPTP-HCl dosage used in the present study was 8 mg/kg (free base) whereas Natale et al. (2010) employed a higher dose of 20 mg/kg, despite using the same mouse background. These differences in both anatomical focus and neurotoxin dosage likely contribute to the more pronounced loss of TH^+^ submucosal neurons reported by Natale et al. (2010).

**Figure 7:**
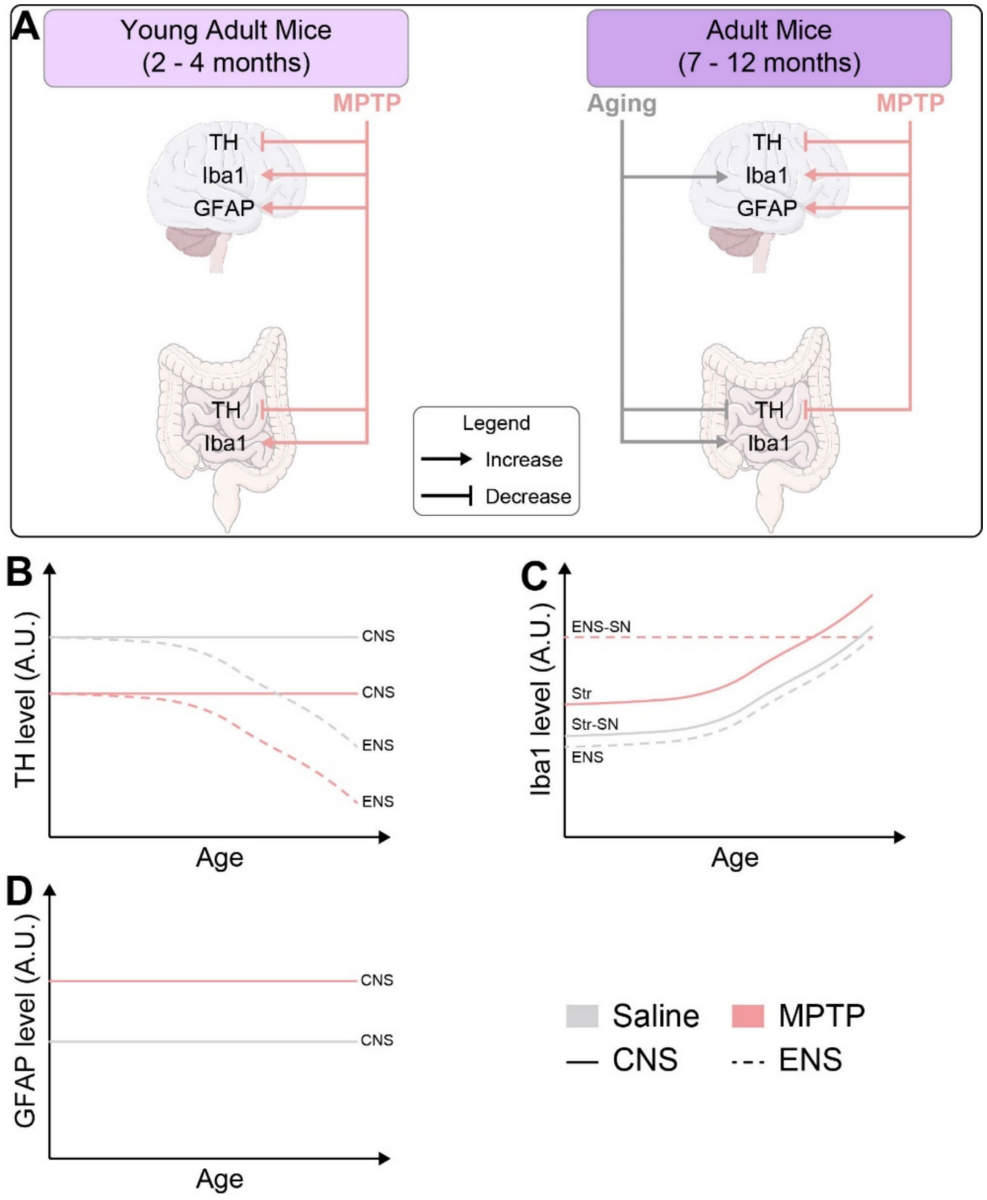
**(A)** Schematic representation of the impacts of aging and MPTP treatment on the enteric and central nervous systems as observed in our study. **(B)** Time course representation of TH levels in mice treated with MPTP vs. saline in the CNS and ENS. **(C)** Time course representation of Iba1 levels in mice treated with MPTP vs. saline in the CNS and ENS. **(D)** Time course representation of GFAP levels in mice treated with MPTP vs. saline in the CNS and ENS. TH, tyrosine hydroxylase; Iba1, ionized calcium-binding adapter molecule 1; GFAP, glial fibrillary acidic protein; CNS, central nervous system; ENS, enteric nervous system; SN, substantia nigra; Str, striatum.

A recent study also proposes that the adult ENS is maintained in a dynamic balance between apoptosis and neurogenesis, replacing old connections with new ones (Kulkarni et al., 2017). In the present study we also report macrophage infiltration in the myenteric plexus during aging, at similar levels to what is observed in mice receiving MPTP injections. We noticed that aging contributes to neuroinflammation and neurodegeneration, particularly within the ENS before impacting the CNS (Figure 7) and that the inflammaging observed appears more pronounced in the ENS, indicating a differential progression of neuroinflammation between peripheral and central systems. This early onset of inflammation in the ENS may underlie the prodromal phase of PD, with inflammaging potentially starting in the ENS and extending to the brain, as suggested by the weak and early microgliosis, altered immune responses and oxidative damage in the CNS.

The interaction between aging and PD involves an imbalance between the protective and damaging actions of astrocytes and microglia on neurons (Streit et al., 2004; Rodriguez et al., 2015). Microglial activation seems to be context-dependent (Vilhardt, 2005), with acute activation potentially offering neuroprotection through neurotrophic factor production and synaptic plasticity (Cullheim and Thams, 2007), while chronic activation typically results in neurotoxicity via pro-inflammatory cytokines and free radicals (Kim and Joh, 2006). Microglia play a crucial role in PD pathology, with their presence near degenerating DAergic neurons suggesting a role in initiating neurodegeneration (McGeer et al., 1988). Therefore, the aging-dependent microgliosis observed in the present study could precede DAergic neurodegeneration. Astrocyte activation varies among PD patients, with some showing neuroprotective functions through antioxidant production, anti-inflammatory cytokines, and trophic factors, while others exhibit neurotoxic effects post-cytokine exposure (Damier et al., 1993; Chao et al., 1996; Mirza et al., 2000; Teismann and Schulz, 2004). The chronic inflammatory state in aging animals observed in our study support the notion that ongoing age-related inflammation exacerbates neurodegenerative processes in PD (Hirsch et al., 1998). Hence, the age-dependent astrogliosis observed here could be a response to age-related degenerative changes. The increase in inflammation suggests that the immune system’s response is altered with aging, contributing to the prodromal phase of PD.

## 5 Conclusion

The present study suggests that inflammaging initially affects the ENS and subsequently extends to the brain, in accordance with the progression of PD from the gastrointestinal tract to the central nervous system. Early dysfunctions in the enteric immune system, likely due to immune overactivation, and common features between PD and senescence, such as loss of DAergic neurons and ENS inflammation, emphasize the importance of incorporating the effect of age in studying PD. Studying the early stage of the disease is crucial for understanding the prodromal phase of PD and investigating the etiology in EOPD patients. This supports the notion that prodromal motor and non-motor features of PD result from the combined effects of aging, failure of normal cellular compensatory mechanisms in vulnerable brain regions, genetic risk factors and specific lifestyle, nutritional, and environmental determinants, including exposure to potentially toxic substances (Hindle, 2010; Calabrese et al., 2018).

## Data Availability

The raw data supporting the conclusions of this article will be made available by the authors, without undue reservation.

## Supplementary material

SUPPLEMENTARY FIGURE 1 Effect of age and MPTP treatment on TH+ neurons in the submucosal plexus. TH+ neuronal population density in the submucosal plexus of saline and MPTP treated mice. Values shown are the mean cell count /mm2 tissue area ± SEM (7-11 mice per group). No significant difference was observed using a 2-way ANOVA, Tukey test.
