# Supplementary figures and images for: Impact of aging on the central and enteric nervous system in a Parkinson’s disease mouse model
Source: Front Aging Neurosci. 2025 Dec 1;17:1576325. doi: 10.3389/fnagi.2025.1576325 (PMC12702875; doi:10.3389/fnagi.2025.1576325)

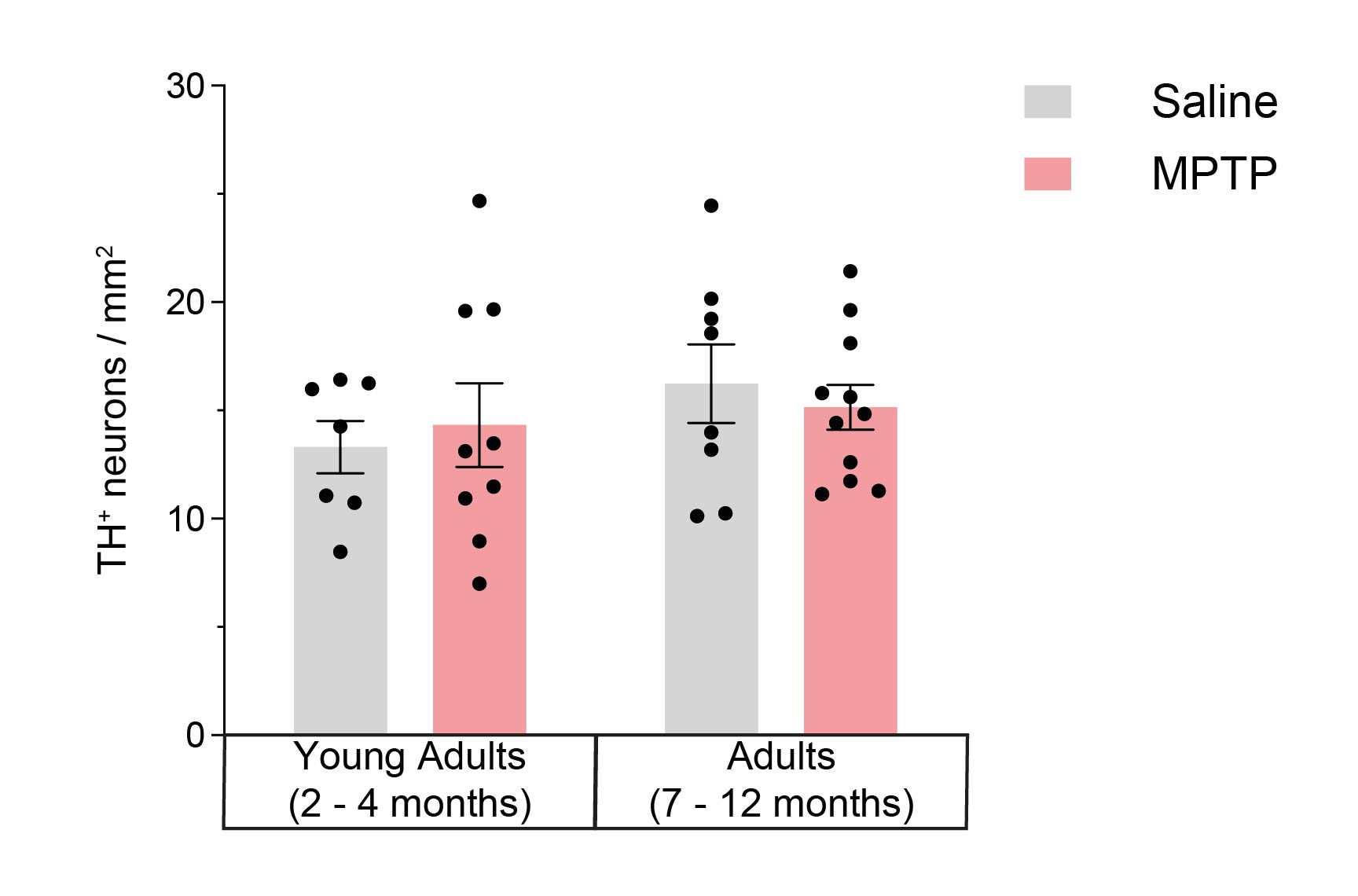

Supplement: SUPPLEMENTARY FIGURE 1 — Effect of age and MPTP treatment on TH+ neurons in the submucosal plexus. TH+ neuronal population density in the submucosal plexus of saline and MPTP treated mice. Values shown are the mean cell count /mm2 tissue area ± SEM (7-11 mice per group). No significant difference was observed using a 2-way ANOVA, Tukey test. [file Image_1.JPEG]
